# Knowing the fishery to know the bycatch: bias-corrected estimates of harbour porpoise bycatch in gillnet fisheries

**DOI:** 10.1098/rspb.2022.2570

**Published:** 2023-07-12

**Authors:** Lotte Kindt-Larsen, Gildas Glemarec, Casper W. Berg, Sara Königson, Anne-Mette Kroner, Mathias Søgaard, David Lusseau

**Affiliations:** ^1^ DTU Aqua, National Institute for Aquatic Resources, Technical University of Denmark, Kemitorvet 1, 2800 Kongens Lyngby, Denmark; ^2^ SLU, Department of Aquatic Resources, Swedish University of Agricultural Sciences, Lysekill 45330, Sweden

**Keywords:** fisheries sustainability, bycatch, harbour porpoise, gillnet, SDG14, SDG15

## Abstract

Incidental captures (bycatch) remain a key global conservation threat for cetaceans. Bycatch of harbour porpoise *Phocoena phocoena* in set gillnets is routinely monitored in European Union fisheries, but generally relies on data collected at low spatio-temporal resolution or over short periods. In Denmark, a long-term monitoring programme started in 2010 using electronic monitoring to collect data on porpoise bycatch and gillnet fishing effort at a fine spatial and temporal scale, including time and position of each fishing operation, together with every associated bycatch event. We used these observations to model bycatch rates, given the operational and ecological characteristics of each haul observed in Danish waters. Data on fishing effort from the Danish and Swedish gillnet fleets were collected to predict fleet-wide porpoise bycatch in gillnets at regional level. Between 2010 and 2020, yearly total bycatch averaged 2088 animals (95% Cl: 667–6798). For the Western Baltic assessment unit, bycatch levels were above sustainability thresholds. These results demonstrate that fishing characteristics are key determinants of porpoise bycatch and that classical approaches ignoring these features would produce biased estimates. It emphasizes the need for efficient and informative monitoring methods to understand possible conservation impacts of marine mammal bycatch and to implement tailored mitigation techniques.

## 1. Introduction

Incidental and unintended catches, often called bycatch, remain the dominant global threat for the conservation of marine mammals and other protected and threatened marine species [1]. Gillnet fisheries are one of the key contributors to marine mammal bycatch [2,3]. Yet, in comparison to other gears, gillnets are generally considered attractive solutions to minimize environmental impacts associated with fishing [4]. Gillnet fishing is a key contributor to small-scale fisheries globally, including in Europe, hence disproportionally contributing socioeconomically to coastal community livelihoods [5]. It is therefore crucial to find ways to reduce the gillnet biodiversity footprint to ensure food security, while fulfilling increasingly stringent environmental legislations [6,7]. We need efficient monitoring methods to manage marine mammal conservation bycatch impacts and to mitigate fisheries characteristics associated with elevated bycatch rates.

Estimating the bycatch of marine mammal species in an area requires fishing effort estimates for the fishing fleet(s) responsible for incidental captures and their respective bycatch rate estimates. Such data need to be representative of both fleet effort and bycatch rates, e.g. using randomized sampling stratification [8]. Estimates of marine mammal bycatch exist locally or regionally [9–11], but are frequently based on partial datasets of effort, bycatch and/or species distribution [12]. Bycatch data collection is often limited qualitatively (e.g. non-representative fleet sample, partial fishing effort coverage, lack of accurate information on bycatch events, etc.) and quantitatively. These uncertainties and lack of accuracy propagate to yield bycatch estimates with large uncertainties and potential biases, decreasing their value for management [13].

Bycatch rates in gillnets are dependent on a combination of biotic and abiotic factors such as the spatio-temporal distribution of the sensitive species and the target species, and the characteristics of the fishing gear (e.g. mesh-size, net height, net-length and soak-time) [14]. The contribution of these factors to bycatch mortality remains broadly unassessed, whereas such knowledge is a key to selecting appropriate mitigation schemes and to shaping up future bycatch-safe fishing gears.

The harbour porpoise (*Phocoena phocoena*) is an abundant cetacean categorized globally as ‘Least Concern’ on the IUCN Red List [15]. Yet, the species is subjected to high bycatch rates in coastal gillnet fisheries throughout its range, leading to conservation challenges for several populations. In Europe, the harbour porpoise is for example listed as Vulnerable [16], while the status of the populations in the Baltic Sea and in the Black Sea is more concerning (Critically Endangered and Endangered, respectively) [17,18]. Regionally, the Belt Sea—a region situated between Denmark and Sweden—is occupied by a management unit classified as vulnerable, but using different criteria from the IUCN Red List classification [19]. The Danish North Sea coast and the West Baltic Strait (Kattegat, Skagerrak and Belt Sea) are both areas of high harbour porpoise densities [20], while also being important fishing grounds for commercial gillnet vessels. There are therefore high risks of bycatch in the region that can affect two key European management units of the species.

In Northern Europe, evaluating the magnitude of harbour porpoise bycatch in gillnets fisheries has been a subject of attention for several decades [21–24]. Vinther [25] estimated that at least 6785 porpoises (CV = 0.12) were taken as bycatch annually between 1994 and 1998 in the North Sea Danish set net fishery, using a combination of fisheries observer data and official landings declarations. More recently, electronic monitoring (EM) systems with video have shown great potential to monitor and estimate bycatch of air-breathing species in gillnet fisheries, including harbour porpoise [26–30]. These EM systems provide detailed fine-scale information for all fishing operations (setting and hauling) over extended periods. Since 2010, volunteering Danish commercial gillnetters have been equipped with EM systems. Detailed EM data offer a unique opportunity to analyse the factors that affect the variance in bycatch rates of harbour porpoise and can be used to model bycatch rate and to estimate fleet-wide bycatch levels using appropriate statistical tools [31,32]. Ultimately, understanding the bycatch levels to which harbour porpoises are exposed regionally is crucial to ensure an effective management of the fisheries operating in the North Sea and the Baltic Sea.

Here, we use one of the longest EM bycatch observation series in the world to develop a model describing the variance in bycatch rates associated with fisheries characteristics. We then use this model to estimate annual bycatch levels to which the Kattegat-Belt Sea harbour porpoise management unit has been exposed and appraise whether these levels are sustainable. Finally, we discuss the insights from the model to understand fishing characteristics that could be managed to reduce bycatch.

## 2. Materials and methods

### (a) Data sources

#### (i) Electronic monitoring data

**Figure 1.**
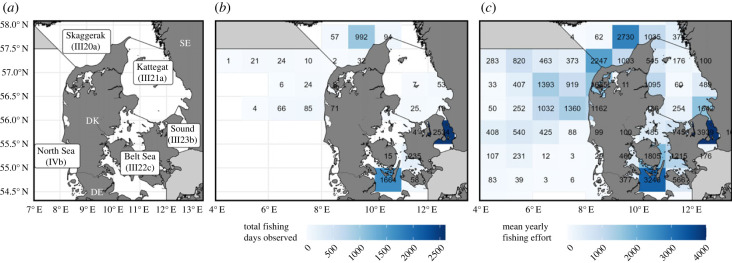
Comparison between EM sampling effort and fishing effort of the Danish and Swedish commercial gillnet fleets. Map (*a*): study area with country codes and ICES statistical area names; map (*b*): number of fishing days observed with EM in the Danish commercial gillnet fleet; map (*c*): average yearly fishing effort of the Danish and Swedish commercial gillnet fleets (as mean number of fishing days per ICES statistical rectangle per year, excluding trips with mandatory acoustic deterrent use according to EU regulations EC814/2004 and EU2019/1241). Regions in grey are outside the study area. Fishing effort data from the German fleet are not included. Data from 2010 to 2020.

Bycatch of harbour porpoises in commercial gillnet fisheries in Denmark and Sweden was estimated using EM systems installed onboard 17 Danish gillnet vessels between 2010 and 2020. Individual vessels' monitoring varied from several consecutive months to years. The sampling area covered the most important commercial gillnet fishing grounds around mainland Denmark and Western Sweden, however not including the Baltic Proper (figure 1*a*).

Monitoring was conducted using two different EM systems. EM Observe (Archipelago Marine Research, Canada, https://www.archipelago.ca/) was used on all participating vessels from 2010 and replaced in 2013 with Black Box Video (Anchorlab, Denmark; http://www.anchorlab.dk/). Both EM systems consisted of a control unit, associated with a position sensor (GPS) and a set of at least two waterproof CCTV (closed-circuit television) cameras recording the fishing activity. The cameras were positioned to allow (by)catch items to be observable from different angles—where the net appears from the water and at the sorting table—thus maximizing the chance of identifying target and bycatch species with certainty.

Each EM system came with its own analyser programme—respectively, EM Interpret (Archipelago, Marine Research) and Blackbox Analyzer (Anchorlab). These programmes show a map with the GPS trace of a vessel, alongside the videos. Trained EM analysts manually reviewed a census of the EM data to detect fishing events and bycatches. In short, EM data processing consisted of identifying each fishing event (setting and hauling of nets) and reviewing the entirety of the video data for each haul to spot harbour porpoise bycatches. Each bycatch event was marked with a timestamp recording location and time of day. Both EM analysing softwares could replay a sequence, adjust playback speed or zoom on areas of interest during the review process. Video quality could vary greatly depending on e.g. darkness, weather conditions or the general cleanliness of the camera lenses. Exceptionally, videos' feed quality was too bad to detect a porpoise bycatch event, and on such occasions ‘true’ bycatch rates would have been underestimated. However, based on the small amount of low-quality data, we considered this negligible. All EM data were saved to be reviewed multiple times if needed. A detailed protocol is available online (see https://www.dcf-denmark.dk/-/media/sites/dcf/manuals/blackbox-analyzer-protocol.pdf).

### (b) Fishing fleet effort data

In the European Union (EU), the fishing activity of all vessels above 10 m—the limit is down to 8 m in the Baltic Sea if the main target is cod (*Gadus morhua*)—must be registered in daily-reported logbooks [33,34], but local disparities exist between Member States. In Denmark, logbook data contain information on the type of fishing gear, the date of fishing operations, the fishing location at the scale of ICES statistical rectangles and the weight of retained catches for each target species. Danish vessels below 10 m (or 8 m if targeting cod in the Baltic Sea) fill in monthly landing declarations in which they report total monthly catches for each species per gear type per ICES rectangle. Moreover, sales notes provide additional information on the species-specific landings weight for each fishing trip, which can be used to estimate the corresponding number of fishing days in the area at the individual vessel level. Swedish commercial fishers are required to fill in more detailed daily logbooks for each fishing operation for all vessels over 10 m (8 m if the main target is cod). Swedish daily logbook data contain information on the type of fishing gear, the date of fishing operations, the fishing location, the weight of retained catches for each target species, and on the fishing effort in metres of net and soak-time in hours for each fishing gear. Like Danish vessels, Swedish commercial vessels below 10 m (8 m if the main target is cod) are required to report effort in monthly declarations, including a summary of the monthly catch in weight per species and the fishing locations, together with the sum of the length, average soak-time and mesh-size of the net-fleets.

A census of the Danish and Swedish fisher-reported data, including all official logbooks, monthly declarations, sales notes and landing declarations, was collected for all the commercial vessels having registered set nets as their primary or secondary gear between 2010 and 2020 (figure 1*c*). Although there are differences in reporting catches and landings between Denmark and Sweden, the gillnet fisheries in these countries share a common fishing ground and are largely similar in their fishing patterns, so we assumed that observations made in the Danish fleet of porpoise bycatch could be applied to Swedish vessels. Importantly, information from German gillnetters who also operate in the western part of the Baltic Sea was not included, as German gillnet fishing effort data are not reported at a comparable spatial and temporal scale [35].

Danish and Swedish effort datasets were cleaned for clerical errors (e.g. fishing locations, non-gillnet gears, etc.). We summarized the data, so that one row would correspond to a unique fishing day per ICES rectangle per vessel. When more than one mesh-size was reported for the same fishing day in the same ICES rectangle, only the mesh-size of the main target species (in total weight of the landings) was kept. Electronic supplementary material, figure S1 summarizes the changes in fishing effort by mesh-size class. Soak-time was averaged out from the values reported in each stratum and net-length was calculated as the sum of all the net fleet lengths in each stratum. In the Danish data, soak-time and net-length were only readily available for the vessels monitored with EM. Retrieving these data was nevertheless essential to run the subsequent analytical modelling, so we used the vessels in the EM dataset as a reference fleet, estimated the average net-length and soak-times from the validated EM data and applied these values to the portion of the Danish fleet not monitored with EM, using expert judgement in the strata with low sampling effort (electronic supplementary material, table S1). Smaller vessels for which only monthly declarations exist do not report their fishing locations, thus their fishing effort was allocated to the ICES rectangle in which their home harbour lies. Depth was estimated for each point in the datasets using a high-resolution bathymetric map and *ad hoc* functions in the R statistical software [36]; for Danish vessels, these corresponded to the mean depth of the ICES rectangle in which fishing had been recorded. Following this, the effort data from the Danish and Swedish fleets were subset to the areas where porpoise bycatch data had been collected with EM, i.e. the North Sea, the Skagerrak, Kattegat, the Belt Sea and the Sound (ICES areas IVb, IIIa20, IIIa21, IIIc22 and IIIb23, respectively). The two datasets were then merged into one containing both Swedish and Danish data. Finally, in order to explore the effect of the current acoustic deterrent (pinger) regulations on total bycatch, we created a second effort dataset, where all the fishing trips for which the use of a pinger was mandatory according to the European legislation [37,38] were removed, under the assumption that no porpoise bycatch occurred during these trips. We explore the impact of this assumption in §3.

### (c) Porpoise bycatch rates and total bycatch

The total number of harbour porpoise bycaught in the Danish and Swedish commercial gillnet fleets was estimated in two alternative ways. First, we estimated mean observed bycatch rates, stratified spatially and temporally, using EM data collected on Danish vessels (bycatch per unit effort, BPUE) and raised these numbers to the number of fishing days in the fleet to estimate total bycatch. Next, we used the same EM data to build a statistical model to predict bycatch of porpoise from operational variables in the entire fleet and ecological variables in the surveyed areas. The results from these two approaches were then compared in terms of mean estimates and uncertainty.

#### (i) Stratified mean estimator

Scaling-up observed bycatch rates to fleet-level is often used in bycatch studies [8]. Here, we estimated mean quarterly bycatch rates for each ICES area from EM data (expressed as the number of porpoises per ICES rectangle per fishing day) and multiplied these with the effort in the corresponding strata taken from logbook and sales notes data (expressed as fishing days per ICES rectangle). The confidence intervals around the point estimates were calculated using a non-parametric bootstrap (100 000 replicates).

#### (ii) Model-based approach

Mixed models allow for departure from independence in ecological data [31,32]. Here, a generalized linear mixed model (GLMM) was developed from EM observations to estimate total bycatch of porpoise at the fleet-level from model predictions. The response variable was defined as the number of porpoises captured per fishing day per ICES rectangle per vessel and was modelled as a count. An important constraint for building models was that only variables that could be retrieved from the Danish and Swedish fisher-reported effort data (logbooks, sales notes and monthly declarations) could be included. We established a list of parameters to test in the candidate models, including operational and ecological variables (table 1). Initially, a full model with all the fixed variables susceptible to contribute to bycatch was created, using as random intercept the unique vessel identification number. Moreover, assuming that the closer in space, the more similar the observations, we added a spatial autocorrelation parameter to each candidate model with an exponential covariance structure. To account for the non-independence of the observations in time, we added a temporal component to the autocorrelation parameter. Preliminary investigations showed that the data were overdispersed and a negative binomial response distribution with a log link was preferred to account for this.

**Table 1.** Summary of the fixed and random variables used in the candidate models.

response variable		
porpoise bycatch	number of porpoises captured per fishing day per ICES statistical square	
fixed effects		
mesh-size	stretched mesh-size (mm) in the conducted fishery, based on fisher declarations or deducted from landings composition	discrete (3 levels): ‘<120 mm’, ‘120–200’, ‘>200 mm’
vessel-length	total length (m) of the fishing vessel	discrete (5 levels): ‘<8 m’, ‘8–10 m’, ‘10–12 m’, ‘12–15 m’, ‘>15 m’
population	dummy variable indicating the porpoise (sub)population, based on the fishing location	discrete (2 levels): ‘North Sea Population’, ‘Western Baltic Population’
net-length	total (log) length of the net-fleets (m) for each fishing day (electronic supplementary material, table S1)	continuous
soak-time	mean (log) soak-time of the net-fleets (hours) for each fishing day (electronic supplementary material, table S1)	continuous
depth	mean (log) depth of the ICES rectangle in which the fishing operation(s) occurred	continuous
random effects		
vessel id	fishing vessel unique identifier	discrete (17 levels)
year	year during which fishing occurred	discrete (11 levels)
spatial autocorrelation		exponential covariance structures estimated independently for each combination of quarter, year, or quarter among year (up to 44 levels)

Alternative models were compared using the Akaike Information Criterion modified for small sample size (AICc). All data treatments and analyses were conducted in R, using the *glmmTMB* package to create the models and the *bblme* package to compare them [39,40]. The selected model was checked for misspecifications and goodness of fit using simulation tools from the *DHARMa* package [41] (electronic supplementary material, figures S4–S13). Lastly, the parameter estimates from the model were used to calculate porpoise bycatch estimates on the natural scale with their associated uncertainty from the combined Danish and Swedish fisher-reported dataset (function *predict* in R). This was done using a modified version of the *glmmTMB* package that included an *aggregate* function (https://github.com/glmmTMB/glmmTMB/tree/aggregate), which allowed both quantification of the uncertainty and the performance of bias correction for sums of predictions on the natural scale (as opposed to the link scale, which is logarithmic). Bias correction is necessary because sums on the natural scale are a nonlinear transformation of the random effects in the model [42]. Corresponding R scripts are publicly available (https://github.com/gildas-glemarec/bias-corrected-estimates-of-harbour-porpoise-bycatch-in-gillnet-fisheries).

### (d) Standardized fishing effort and standardized bycatch rate estimations

As opposed to the scaling-up approach, a model-based method could account explicitly for variations in operational factors in the gillnet fishery, e.g. differences in vessel-length, mesh-sizes, or soak-time. In turn, we could explore the underlying reasons for possible trends by standardizing bycatch rates and fishing fleet effort. For this, the terms in the model formula were grouped in two categories. One group described the effect of different types of fishing effort on the amount of bycatch, i.e. vessel, mesh-size and soak-time effects, while the other group described changes in porpoise density over time and space. This separation of model terms allowed for calculating not only standardized bycatch numbers, but also standardized effort and porpoise BPUE relative to other years/areas.

The standardized effort was calculated by fixing all porpoise density terms in the model to some arbitrary constant value for all years and spatial positions (e.g. the median value), and predicting the amount of bycatch given the observed effort data. The result was then proportional to the bycatch per individual porpoise for a given set of effort data, since it accounts for different values of e.g. mesh-size, soak-time and net-length having different risks of catching porpoise. Similarly, the standardized BPUE was calculated by fixing all the effort-related variables to constant values and predicting the bycatch for each year in all spatial positions.

$$\begin{array}{rl} \mathrm{porpoise}\;\mathrm{bycatch} & =\exp(\underset{\mathrm{effort}\text{-}\mathrm{related}}{\underbrace{\mathrm{mesh}+\mathrm{soak}+\mathrm{net-length}+(\ldots )}}\\ & \;+\underset{\mathrm{porpoise}\text{-}\mathrm{related}}{\underbrace{\mathrm{spatio-temporal}}}). \end{array}$$

This separation of effort and porpoise density terms offers a possibility to produce spatially explicit estimates of relative porpoise densities (standardized BPUE) and relative porpoise mortality due to fishing (standardized effort) in addition to total bycatch estimates. The total bycatch is consequently the product of porpoise density and effort in each stratum (electronic supplementary material, figure S4). Moreover, what we refer to as ‘standardized effort’ is not necessarily indicative of the actual fishing effort but is rather the porpoise mortality induced by the fishing effort.

### (e) Sustainable bycatch limits for the Western Baltic assessment unit

We estimated the potential biological removal limit (PBR) for harbour porpoise in the Western Baltic (encompassing ICES areas IIIa21, IIIb23 and IIIc22), to evaluate the sustainability of the bycatch levels in that assessment unit [43]. There is no clear quantification of the conservation objective to achieve in European waters. Several Conventions (e.g. ASCOBANS) have interpreted the European Commission's regulation to ‘minimize and where possible eliminate [bycatch] such that they do not represent a threat to the conservation status of these species' [37] to mean that Member States should aim to see cetacean populations *recovering* to 80% of their carrying capacity in the long-term [44]. This has been interpreted as a tuning objective for PBR to mean that during robustness trials, the objective was to define PBR so that 80% of simulated populations reached or stayed above 80% of populations' carrying capacity over 100 years. Some parameter values can be considered to achieve this objective when retuning PBR into what has been called *modified* PBR (mPBR) [45]. Here, we estimated PBR using two alternative approaches. First, we used the preestablished parameter values from Genu *et al*. [45] to tune PBR (mPBR) to the Western Baltic porpoise population assessment unit. Then, we estimated the PBR-informed threshold using the common approach where bycatch limits are estimated based on the management goals of the US Marine Mammal Protection Act [43]. This classical approach aims to ensure that during robustness trials, 95% of the simulated populations are not depleted, i.e. stay at or above 50% of carrying capacity over 100 years. Porpoise population abundance in the Western Baltic was previously estimated using density estimation methods [20]. However, the area surveyed to estimate these densities varied between the three surveys that took place during this paper's study period (2012, 2016 and 2020). We therefore re-estimated abundances based on each respective density estimate using the same surface areas as the one sampled for bycatch, i.e. the Kattegat (IIIa21), the Sound (IIIb23) and the Belt Seas (IIIc22), covering respectively 20 536 km^2^, 2281 km^2^ and 17 862 km^2^. Then, we estimated four threshold values (two PBR- and two mPBR-based) for each year in which an abundance estimate was available, with recovery factor values associated with uncertain estimation processes for abundance and bycatch, and with more accurate and precise estimates (respectively, recovery factor (*F*_r_) = 0.5 or 1.0 for PBR, and *F*_r_ = 0.15 or 0.35 for mPBR).

## 3. Results

In total, 6139 individual fishing days were recorded and analysed in the Danish EM fleet between 2010 and 2020, during which 525 harbour porpoise bycatches were observed in hauls where no pingers were used. At least one porpoise was registered as bycatch in 8.6% of the recorded fishing days.

### (a) Model selection

The model selection (electronic supplementary material, table S2) favoured a spatial autocorrelation component structure with a random variation of exponential decay in correlation with the distance within year and within quarter. This indicates that bycatch observations tend to cluster both in space and in time or, in other words, that there exist areas of higher risk of bycatch. The random variable coding for the unique vessel identifier (*id*) was not retained in most models, suggesting that the bycatch rates depend foremostly on the characteristics of the fishing gears described by the fixed effects rather than on specific fishing vessel behaviour. Interaction terms generally increased model fit, with the final model retaining only the interaction between mesh-size and soak-time.

The estimates of the regression parameters for the selected model (electronic supplementary material, table S3) indicated that the most prominent contributors to porpoise bycatch were mesh-size, soak-time and vessel-length.

### (b) Porpoise bycatch rates and total bycatch

**Figure 2.**
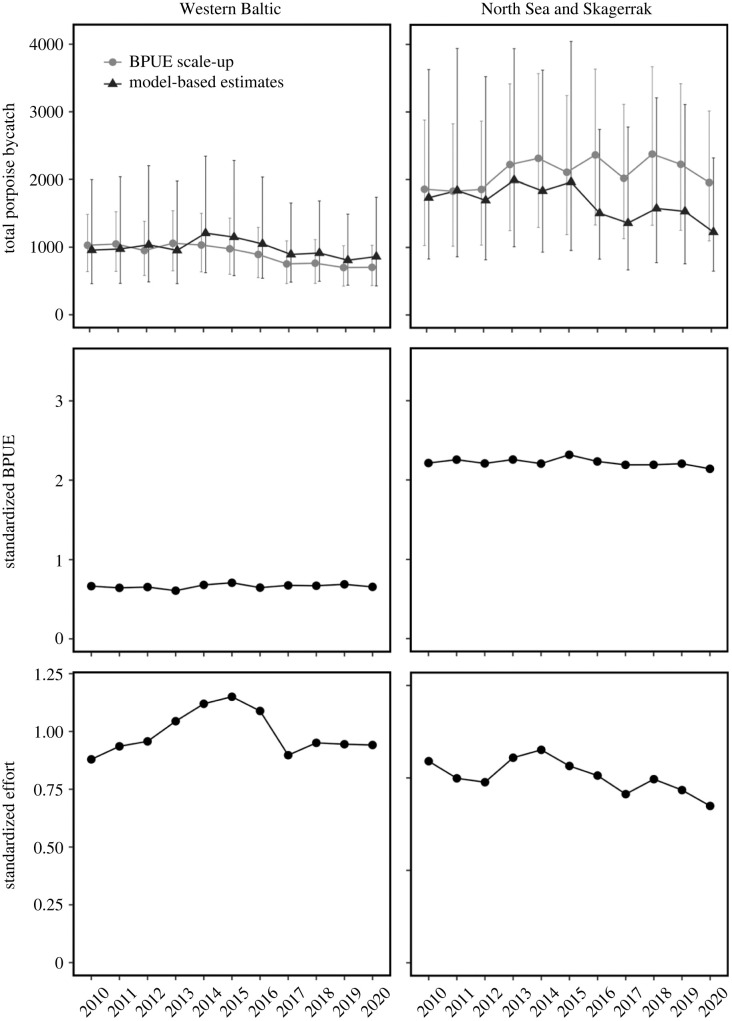
Comparison between total porpoise bycatch including 95% confidence intervals using two alternative estimation methods (top), concomitant standardized bycatch rates (middle) and standardized fishing effort (bottom), in the Western Baltic (left column) and the North Sea & Skagerrak (right column) for 2010–2020.

Total porpoise bycatch estimates from model predictions were aggregated by year for two focal areas: the Western Baltic, and the North Sea and Skagerrak. These numbers were compared with the bycatch estimates obtained from scaling-up the mean EM observed bycatch rates to the entire fleet for the same two focal areas (figure 2—top). Using the bycatch model, we fixed some parameters to constant values to produce standardized porpoise density estimates (figure 2—middle) and standardized fishing effort estimates for each focal area (figure 2—bottom). Note that these are relative estimates (because the choice of constant is arbitrary), so they can only be used to compare different years or areas relative to each other, therefore the scaling on the *y*-axes is arbitrary in the middle and bottom plots in figure 2.

Mean values of the bycatch estimates differed between the two methods (figure 2—top). On average, in the Western Baltic, total bycatch estimates from model predictions were slightly higher than those estimated by scaling-up bycatch rates to the entire fleet, while model-predicted bycatches were considerably lower in the North Sea and Skagerrak compared to the alternative method, especially from year 2016 and onward, where bycatch estimates were almost halved. However, as the model-based approach integrates the variability of the fishery and of the porpoise density, we considered the model-informed estimates closer to the reality. Figure 2 (middle) also shows that the mean porpoise density (BPUE) is more than double in the North Sea and Skagerrak compared to the Western Baltic, whereas porpoise bycatch mortality (figure 2—bottom) is more comparable between areas, but greatest in the Western Baltic, at least in the most recent years.

Standardization demonstrated that, although there are important differences between the Western Baltic and the North Sea and Skagerrak focal areas, with higher mean bycatch rates in the latter (figure 2—middle), the main driver of the temporal bycatch development in the region is the fishing effort of the gillnet fleet (figure 2—bottom). That is, changes in the characteristic of the operational factors of the fleet over time (here, mesh-size, vessel-length, net-length and soak-time) did not significantly affect the mean bycatch rates. Conversely, a reduction in fishing effort intensity (measured as number of fishing days per ICES rectangle) led to a comparable reduction in porpoise bycatch regionally.

**Figure 3.**
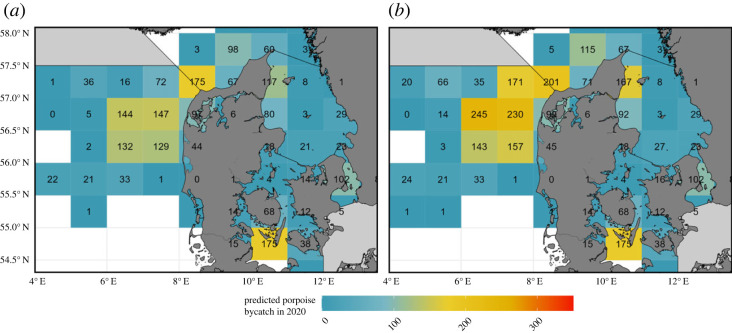
Predicted yearly porpoise bycatch per ICES statistical rectangle for the Danish and Swedish commercial gillnet fleets. Map (*a*): pinger usage following EU regulation 2019/1241 and assuming 100% pinger efficacy; map (*b*): ignoring potential bycatch reduction from pinger usage. Data from 2020.

Model-based estimates revealed areas and times of the year with higher risks of porpoise bycatch (table 2) and allowed for assessment of the potential effect of the Danish–Swedish pinger legislation on porpoise bycatch (figure 3), using effort data from the entire fleet—i.e. assuming that pingers are not used at all—and using a subset of the effort dataset where all fishing days where pinger usage is mandatory were removed—i.e. assuming full compliance of the pinger regulation and that pingers effectively reduce bycatch by 100%. Areas west and northwest of Denmark (in the North Sea and Skagerrak) and areas south of the island of Fyn and in the Sound (in the Baltic sea) were found to be the most problematic in terms of porpoise bycatch.

**Table 2.** Comparison between quarterly and yearly porpoise bycatch per ICES area and per focal area (in bold), ignoring potential bycatch reduction from pinger usage (light grey) and assuming 100% bycatch reduction when pingers are used following the current regulations (white). Mean estimates and 95% bootstrapped confidence intervals (100 000 replicates) from model predictions are stated. Data from the Danish and Swedish commercial gillnet fleets for year 2020.

	area	quarter 1	quarter 2	quarter 3	quarter 4	year
North Sea & Skagerrak	Skagerrak (IIIa20)	14 (4–47)	149 (43–514)	53 (18–154)	40 (12–130)	255 (77–845)
	Skagerrak (IIIa20) no pinger	14 (4–49)	149 (43–516)	84 (28–255)	46 (14–157)	294 (89–977)
	North Sea (IVb)	166 (61–453)	441 (183–1065)	282 (85–933)	82 (22–304)	972 (351–2755)
	North Sea (IVb) no pinger	167 (61–453)	465 (193–1120)	669 (204–2192)	97 (29–332)	1398 (487–4097)
	**total porpoise bycatch**	**180** **(65–500)**	**590** **(226–1579)**	**335** **(103–1087)**	**122** **(34–434)**	**1227** **(428–3600)**
	**total porpoise bycatch no pinger**	**168** **(65–502)**	**614** **(236–1636)**	**753** **(232–2447)**	**143** **(43–489)**	**1678** **(576–5074)**
Western Baltic	Kattegat (IIIa21)	269 (61–1182)	103 (25–413)	48 (16–148)	8 (2–32)	428 (104–1775)
	Kattegat (IIIa21) no pinger	341 (26–1656)	103 (26–413)	53 (18–155)	8 (2–32)	505 (72–2256)
	The Sound (IIIb23)	20 (5–85)	23 (5–101)	36 (10–132)	40 (12–136)	119 (32–454)
	The Sound (IIIb23) no pinger	20 (5–85)	23 (5–101)	36 (10–132)	40 (12–136)	119 (32–454)
	Belt Sea (IIIc22)	44 (13–141)	72 (25–211)	116 (39–340)	83 (25–277)	315 (102–969)
	Belt Sea (IIIc22) no pinger	44 (13–141)	72 (25–211)	116 (39–340)	83 (25–277)	315 (102–969)
	**total porpoise bycatch**	**333** **(79–1408)**	**198** **(55–725)**	**200** **(65–620)**	**131** **(39–445)**	**862** **(238–3198)**
	**total porpoise bycatch no pinger**	**405** **(44–1882)**	**198** **(56–725)**	**205** **(67–627)**	**131** **(39–445)**	**939** **(206–3679)**
**all areas**	**total porpoise bycatch**	**513** **(144–1908)**	**788** **(281–2304)**	**535** **(168–1707)**	**253** **(73–879)**	**2089** **(666–6798)**
	**total porpoise bycatch no pinger**	**586** **(109–2384)**	**812** **(292–2361)**	**958 299–3074)**	**274** **(82–934)**	**2617** **(782–8753)**

### (c) Sustainable bycatch limits in the Western Baltic

Given the uncertain nature of the abundance estimates in 2012 and 2016, we selected the thresholds more robust to potential biases in abundance estimates for these years (table 3, values in italics). The abundance estimate for 2020 emerged from the best possible density sampling design, so we decided to use the most certain options to define threshold values for that year (table 3, values in bold).

**Table 3.** PBR and mPBR estimates for the Western Baltic assessment unit, given two assumptions about the robustness of the estimates used to calculate the bycatch limits. Values in bold represent the preferred threshold estimates given the method that was used for estimating porpoise abundance in that year. CV = coefficient of variation; *N*_min_ = minimum population estimate; (m)PBR = (modified) potential biological removal; *F*_r_ = recovery factor.

year	density (individual/km^2^)	area surveyed (km^2^)	abundance (area corrected)	CV	*N*_min_	*R*_max_	PBR *F*_r_ 0.5	mPBR *F*_r_ 0.15	PBR *F*_r_ 1.0	mPBR *F*_r_ 0.35
2012	0.79	51 511	32 136	0.24	26 330	0.04	**263**	**79**	527	184
2016	1.04	40 707	42 306	0.3	33 041	0.04	**330**	**99**	661	231
2020	0.41	42 244	16 678	0.2	14 116	0.04	141	42	**282**	**99**

Porpoise bycatch estimates for 2020 in the Western Baltic focal area (corresponding to the sum of captures in ICES areas IIIa21, IIIb23 and IIIc22) reached 862 animals (238–3198) when assuming that pinger usage strictly follows the legislation and reduces bycatch by 100%, and up to 939 (206–3679) when pinger implementation is ignored (table 2). The sustainable bycatch limit estimates for 2020 shown in table 3 are thus far below the predicted annual bycatch estimates for that year in the Western Baltic area.

## 4. Discussion

To the best of our knowledge, this study is the first to present fleet-level estimates of any cetacean species bycatch in commercial gillnets, correcting for fleet characteristics and using a long time series of fine-scale EM data. This work yields a model able to predict harbour porpoise bycatch from fisheries-dependent data. In the study area—the Eastern North Sea, Skagerrak and Western Baltic—2089 porpoises were predicted to have been bycaught in 2020, provided that acoustic deterrents are used according to the legislation and that they totally mitigate bycatch. Not accounting for pinger usage, we predicted 2617 bycatches in 2020 (table 2). Both estimates are nonetheless substantially less bycatch (about one-third) than reported in the late 1990s [25]. Several factors could have contributed to this difference. First, porpoise density might have decreased substantially since that period, yet population monitoring indicates that density estimates have not changed significantly in this region over this time span [46]. Fishing characteristics could have changed, affecting bycatch rates (fishing effort) or bycatch probability (fishing gear characteristics) [47]. The outcomes of our modelling effort point to fishing effort as a key contributor to changes in total bycatch over the study period (electronic supplementary material, figure S3).

For the Western Baltic, the estimates from model predictions showed very similar results to those using traditional BPUE extrapolation method, likely because the vessels here fish in a very homogeneous way (inshore fishery, day trips, few target species, mostly small vessels). As the fleet is more homogeneous, the monitoring data are also more representative of the fleet, thus the two ways of predicting total bycatch yield similar outcomes. Conversely, the fishery in the North Sea is more heterogeneous, being characterized by a mixture of large and small vessels with varying fishing trip duration, and varying target species needing different net-lengths, mesh-size and soak-times. As the model accounts for these fishing characteristics, the predicted total bycatch estimates emerging from the modelled bycatch rates account for the fleet variability. The BPUE applied to fishing days assumes that all fishing days are equally likely to bycatch porpoises, regardless of fishing characteristics, which can therefore yield biased estimates when the sampling design cannot account for representativeness [8]. This shows the importance of acknowledging fishery variability when trying to estimate fleet-level total bycatch.

### (a) Total bycatch

We estimated that 939 porpoises (206–3679) were bycaught in 2020 in the Western Baltic, when no pingers are assumed to have been used (table 2). This is comparable to previous estimates which estimated that 615 porpoises (360–915) were bycaught annually on average between 2010 and 2018 [29]. However, these estimates ignored the contribution from both the Swedish and German gillnet fleets. Still, both assessments are far above earlier estimates from the ICES Working Group on BYCatch (WGBYC)—which estimated between 165 and 263 porpoise bycatches in the Western Baltic in 2014, yet without accounting fully for the effort of small vessels, which often are underrepresented in national statistics [48]. This last point stresses our conclusion that it is crucial to account for the fishing characteristics in fisheries where porpoise bycatch occurs, not only when estimating bycatch, but also when designing monitoring programmes.

Based on our model predictions, 1678 porpoises (576–5074) were bycaught in gillnets in the Eastern North Sea and Skagerrak in 2020, assuming no pinger usage (table 2). This is lower than previous evaluations, with 5591 porpoise bycatches in Danish gillnets estimated annually in the same area between 1987 and 2001, while these numbers went down to 3887 porpoises in 2001, following reductions in fishing effort intensity [24]. More recently, WGBYC estimated a total annual bycatch of 1175–2126 porpoises in the Greater North Sea (an area larger than what we considered here), which accounted for national fisheries not considered here (e.g. UK) [49].

It is important to recall that, in this paper, German gillnet effort is not included in the estimation process, notably because of the peculiarities of fishing effort reporting in Germany, which is not standardized with other countries [35]. This means that the total bycatch presented here remains an underestimate.

### (b) Spatial and temporal variability in bycatch

Total bycatch is not homogeneously distributed in the study area (figure 3). Areas of high bycatch did not systematically correspond to areas of high fishing effort (figure 1*c*; electronic supplementary material, figures S2 and S3). Instead, predicted high bycatch areas were associated with areas where fishing methods (soak-time, net-length and mesh-size) are more conducive to bycatch. This has important implications for mitigation and shows that simply reducing the number of fishing days in an area of high bycatch may not be sufficient to reduce total bycatch. Our work highlights which fishing techniques should be prioritized for mitigation, once bycatch probability and fishing intensity have been accounted for (electronic supplementary material, figure S3).

Our model prediction approach can help estimate how inferred relative porpoise density, fishing characteristics and fishing intensity interact to yield bycatch levels (electronic supplementary material, figure S3). Standardized bycatch rates (left column in electronic supplementary material, figure S3) is the product of porpoise density and the bycatch probability of individuals. Our model allowed us to separate these two terms and give spatially explicit estimates of relative porpoise density, mortality due to bycatch, and total bycatch as the product of the two. Independent measures of porpoise density are therefore not necessary to estimate spatially explicit measures of relative porpoise densities and bycatch probabilities. The estimated bycatch probabilities capture the spatial heterogeneity in the species propensity to become entangled in fishing gear as the measure is standardized for fishing characteristics. This spatial heterogeneity could emerge from behavioural variance (e.g. individuals paying less attention to fishing gear when foraging) or demographic characteristics (e.g. spatial assortment by age classes). Regardless of causes, a spatially explicit bycatch probability estimate is an extremely useful tool to plan the best-suited mitigation methods.

### (c) Current mitigation measures

The original EU pinger regulation and its succeeding iteration [37,38] have imposed acoustic deterrent usage for the largest vessels only, thereby discounting the contributions of small vessels to bycatch. This decision has received heavy criticism since its implementation (e.g. from ICES working groups and ASCOBANS), arguing that pinger usage should be independent of vessel size. Here, we estimated bycatch levels with and without the implementation of those regulations (in particular, EU2019/1241) assuming that these mitigations are 100% effective, an extremely liberal assumption [50,51]. As such, the estimates shown here provide a conservative estimate—a lower bound—of bycatch without any management errors. This work shows that current pinger implementations have a limited effect on bycatch, and in some cases (depending on region and season) virtually no effect. Indeed, current implementation plans limit pinger usage to a subset of the fleet that in many instances represent at best a small portion of the entire fishing fleet. Few EU Member States impose acoustic deterrents for vessels less than 12 m in length. In Sweden, for those vessels not obligated to use them by law, pingers usage is voluntarily, but encouraged by the fishing authorities and the industry alike. This has led to an industry-led demand, which grew from a few vessels in 2015 to about 25% of the fleet in number of vessels in 2020.

### (d) Limitations and assumptions

Bycatch rate estimation depends on two important assumptions. First, the EM observed data need to be representative of the fleet in terms of area, time of year and fishing characteristics such as vessel-length and target species. Our voluntary EM observation covered most areas except the northern and central part of Kattegat and the southern North Sea (figure 1). The EM coverage has in general reflected seasonal fishing patterns as most vessels are covered all year round and most vessels have been assigned to the programme for years.

All vessels participated voluntarily, and vessel crews were aware that the hauls were being monitored. There is a known tendency for fishers to shift from normal fishing practices when they know they are being observed [52], implying that the average number of porpoises bycaught on an EM vessel could be lower than on an equivalent non-observed vessel. However, the study covers 10 years of data and several of the vessels have been committed to the project during all these years. The chances that vessels would change their fishing patterns to elude bycatch for such a long period are unlikely given the economic impact this would have. It is possible that fishers who perceive that they have high bycatch levels did not sign up for the project. It is thus possible that the numbers presented are underestimates. For the data to be even more representative, one would have to select the vessels to secure data in all classes and then the programme would no longer be voluntary.

When estimating bycatch using our modelling approach, assumptions had to be made about mean net-length and mean soak-time. These assumptions clearly come with errors as no fishers have exactly equal fishing patterns. However, it improves vastly on bycatch estimation methods earlier used by WGBYC, which assumes that all vessels have the same kind of ‘fishing day’ regardless of vessel-length and mesh-sizes. In all cases, this paper stresses the crucial need to have more accurate and precise national statistics on fishing effort. Key variables influencing bycatch probability (soak-time, net-length and mesh-size) are required to derive robust bycatch estimates. In our case, although there are differences in reporting catches and landings between Denmark and Sweden, the gillnet fisheries in these countries share common fishing grounds in the Western Baltic and Eastern Greater North Sea and are largely similar in their fishing patterns, so we assumed that observations made in the Danish fleet of porpoise bycatch and resulting mean bycatch rates could be applied to Swedish vessels.

### (e) The Western Baltic conundrum

Regardless of threshold estimation assumptions, bycatch levels in the Western Baltic are above the threshold values estimated to yield sustainable bycatch limits. Nevertheless, PBR estimates do fall within the confidence intervals of the estimated yearly total bycatch (table 2). Yet the breakdown of total bycatch estimates to their main components appears to indicate that the bycatch rate is constant, and variability is mainly associated with fishing characteristics (figure 2). At the same time, there is no indication of degradation of this population over the studied period, i.e. the trend in abundance is stable over a long period [46].

This could be for two reasons. First, we could assume that the population can sustain the level of bycatch to which it is exposed, implying that the mPBR- and PBR-based threshold values are over-precautious. Regarding mPBR, this makes sense as the objective is to maintain or restore to carrying capacity a population far away from its maximum net productivity level (MNPL). In that state, the net productivity the population can achieve is much lower than MNPL, forbidding sustaining as many excess mortalities. The mPBR objective is to ensure that the population thrives, not that it cannot become depleted. For PBR, however, the objective is that the population remain at or above MNPL with high certainty. Hence, PBR estimates here are likely cautious and should be treated as reference points over which bycatch levels need greater attention rather than a threshold *stricto sensu*. Second, the assessment unit considered here is more open to immigration and/or movement of harbour porpoises from neighbouring regions than previously thought and is therefore ill-defined. Several hundreds of porpoises would need to immigrate yearly into the Western Baltic to maintain a stable population given the discrepancies between the estimated threshold and the level of bycatch predicted from our model. This would mean that the Western Baltic harbour porpoise assessment unit is extremely open to what is currently considered the North Sea assessment unit [46] as the Baltic Proper assessment unit is not large enough to maintain such a level of emigration [53]. In addition, it cannot be excluded that the methodology used to estimate porpoise abundance in the Western Baltic assessment unit underestimates the population size [46]. The underestimation would have to be substantial, to such a level that is unlikely to be the cause given our understanding of line transect sampling methods in these conditions. Likewise, our model could be overestimating total bycatch (see §4d), but the bias would have to be substantial.

Two contrasting conclusions emerge from this work. One option is that we accept the current assessment unit. In this case, PBR is a more appropriate measure of sustainable bycatch limit than mPBR, as PBR falls within the confidence interval of the predicted total bycatch. This would mean that the alternative conservation objective (a restoration objective) first set by ASCOBANS, while well-intentioned, is not useful to guide bycatch management—in this instance at least. Under such a restoration objective, mitigation measures would likely be required from the moment a single porpoise is bycaught. The alternative is that the delineation of the assessment unit is not making sense and we need to consider large influx of porpoises from the North Sea to maintain the population and the bycatch levels observed; in which case the assessment unit needs to be merged with the North Sea one.

## 5. Final remarks

Globally, we need to prioritize the generation of fisheries statistics at a fine scale of effort. This information is crucial as we try to achieve ambitious biodiversity restoration targets while maintaining the vital food security provided by small-scale coastal fisheries [54].

Coastal fisheries are facing rapid socioecological changes, like many other human activities in the 2020s. In the Western Baltic, gillnet fishing effort decreased significantly over the past decade (electronic supplementary material, figure S1), and this will most likely continue due to the current limitations on cod fishing. This will in turn yield a reduction in porpoise bycatch. Nonetheless, as cod gillnet fishers will switch to other target species, change gear, or stop fishing, the way in which changes in fishing practices and intensity will affect porpoise bycatch remains largely unknown. Furthermore, we show here that the current pinger deployment regulations are ineffective at reducing bycatch; not because pingers are not an effective tool to reduce bycatch, but because the regulated pinger deployment schedule is not targeting the vessels and instances yielding the greatest bycatch risk.

## Data Availability

Corresponding R scripts are publicly available (see https://github.com/gildas-glemarec/bias-corrected-estimates-of-harbour-porpoise-bycatch-in-gillnet-fisheries). Data are available on demand. However, some of the data are sensitive and GDPR-protected. Data contain both pictures with faces, vessel names and fishing position data. Thus restrictions are needed, but data can however be made available in summed formats. The data are provided in the electronic supplementary material [55].
